# Physical fitness profile of primary school learners in the Eastern Cape province of South Africa

**DOI:** 10.4102/hsag.v29i0.2611

**Published:** 2024-08-21

**Authors:** Howard Gomwe, Lesego Phiri, Chioneso S. Marange

**Affiliations:** 1 Skills Centre, Faculty of Health Sciences, Sefako Makgatho Health Sciences University, Pretoria, South Africa; 2 Department of Statistics, Faculty of Science, University of Fort Hare, East London, South Africa

**Keywords:** anthropometric measurements, physical fitness, physical activity, BMI, children, rural, urban

## Abstract

**Background:**

Low levels of physical activity in high- to low-income countries, including South Africa, pose a great risk of cardiovascular-related diseases.

**Aim:**

This study aimed to assess and compare the physical fitness profile among children.

**Setting:**

The study setting comprised rural and urban primary school learners in the Eastern Cape province, South Africa.

**Methods:**

A cross-sectional study was adopted utilising a randomly selected sample of boys and girls aged 9–14 years. Physical fitness measures were assessed using the Euro-fit test battery. Some parametric tests were used for mean comparisons of the various anthropometric measurements and physical fitness characteristics across demographics.

**Results:**

The sample consisted of 870 primary school learners. The average weight and height of the sample were 39.29 ± 10.34 kg and 144.06 ± 10.81 cm, respectively, with a mean body mass index (BMI) of 18.80 ± 4.11 kg/m^2^. The results showed that boys reported significantly high levels of physical fitness. Sit-ups (*p* ≤ 0.001) and VO2 max (*p* ≤ 0.001) significantly increase with age, while all the physical fitness measures were significantly higher among rural-based learners.

**Conclusion:**

Rural-based learners and boys generally had better physical fitness performance than their peers. The effect of gender and place of residence should be considered when designing physical fitness interventions.

**Contribution:**

This study adds to the existing body of knowledge about the effect of demographic factors on the physical fitness profile of children, where boys and rural-based learners are reported to be more physically fit than their counterparts.

## Introduction

Globally, the problem of physical inactivity in high-, middle-, and low-income countries, including South Africa, has reached an alarming level (Ammar et al. 2023). Physical inactivity increases the prevalence of levels of non-communicable diseases (NCDs) and metabolic diseases like hyperlipidaemia, hyperinsulinaemia, hypertension, glucose intolerance and obesity in children and adolescents (Chacón-Cuberos et al. 2018; Palou et al. 2019; Sierra-Díaz et al. 2019). On the other hand, physical activity (PA) is one of the main contributing factors to physical fitness (PF) (Calella et al. 2023; Hussey et al. 2007; Ruiz et al. 2006). Therefore, assessing PF levels of school learners is vital to mitigate risk factors associated with NCDs and metabolic diseases (Chacón-Cuberos et al. 2018; Palou et al. 2019; Sierra-Díaz et al. 2019).

Physical fitness is defined as the ability of the body to function effectively and efficiently, enjoy leisure, be healthy, resist disease and cope with emergencies (Hian, Mahmud & Choong 2013). Physical fitness is classified into two categories that have a similar meaning: health-related (health state and well-being) and skill-related (the ability to perform certain aspects of physical activities) (Hian et al. 2013). Health-related components of PF include body composition, cardiovascular fitness, flexibility, muscular endurance and strength. Skill-related components include agility, balance, coordination, power, reaction time and speed (Olds et al. 2006; Ortega et al. 2008; De Smet et al. 2023; Greco et al. 2022). Therefore, the importance of conducting a research study on the status of children’s PF is justified.

In the literature, demographic factors like age, gender and residence (rural and urban) are reported to possibly influence PF (Gomwe et al. 2022). It has been noted that, based on the South African perspective, there has been a gradual decline in both PF levels and activity levels in primary school learners (Nqweniso et al. 2021). This is mainly attributed to the easy access to transport as the unsafe environment, and the distances between schools and residential areas make it difficult to walk to and from school (Gomwe et al. 2022; McVeigh & Meiring 2014; Monyeki 2014). On the other hand, in most circumstances, rural school learners are more active than their urban counterparts because they walk to and from school on foot for long distances (Craig, Bland & Reilly 2013). One of the major reasons is the low crime rates in rural areas compared to urban areas, so learners are free to walk without fear, so their PF levels increase (Craig et al. 2013).

A study was conducted in Malaysia to assess the effect of residence on PF using primary school learners aged 10–13 years (Hian et al. 2013). The study examined strength, flexibility, power and cardiovascular endurance, and the results showed that rural learners were better at health-related fitness than urban school learners. Sylejmani et al. (2019) investigated to determine the differences in PF components among children and adolescents from urban and rural areas in Kosovo. The study’s major aim was to determine the effect of the place of residence on PF of school learners among both boys and girls. The results showed that rural school learners had lower body mass index (BMI) and lower average height than urban school learners. Regarding cardiorespiratory, sit and reach and sit-ups, rural school learners performed better than their urban peers. Chillon et al. (2011) examined the differences in PF components between rural and urban Spanish school learners. Physical fitness levels were assessed by a 20 m shuttle run, speed shuttle run, sit and reach, push-ups (per min) and sit-ups in 30 seconds. The results revealed that rural school children had higher cardiorespiratory fitness, push-ups and sit-ups but lower flexibility than their urban peers.

Joens-Matre et al. (2008) assessed the PF levels between urban and rural children in the United States, North Carolina. Participants (1687 boys and 1729 girls) were recruited from fourth- to sixth-grade classes in schools in urban areas, small cities and rural areas. The prevalence of overweight was higher in urban areas than rural areas. Children in urban areas were more active and had higher levels of PF than rural children. The results of the study suggest there are rural–urban differences in children’s prevalence of overweight and PA. Torres-Luque et al. (2018) examined the effect of place of residence on PF in Spain. The research used 363 schoolchildren, both boys and girls. The results showed that boys performed better in shuttle run tests, push-ups and sit-ups than girls. Children in urban areas were taller than those in rural areas and performed better. The effect of age was larger than the effect of residence in all fitness measurements. The effect of gender was observed more in boys than girls. Gouveia, Forte and Coelho (2021) compared PF levels by gender and residence of school children in Portugal. The results showed that boys performed higher in the push-ups and aerobic capacity tests, whereas girls showed higher values in the sit and reach test. It seems reasonable to assume that there is a sociodemographic influence on PF levels of school learners. It seems also that rural life provides more opportunities for children to be more active than those in urban areas.

Most studies in South Africa have extensively addressed the comparison of body composition and PF by gender and age of children. In the Northwest province, Monyeki et al. (2005) conducted a study with 855 rural participants aged 7–14 years, and BMI was found to have a significantly positive relationship with the sit and reach (cm) test. In another study in the Northwest province, Truter, Pienaar and Du Toit (2010) examined 280 participants aged 9–12 years and reported that the mean values of PF parameters decreased as BMI increased from normal weight to obese. A study by Travill (2007) conducted in the Western Cape province among 720 urban socially disadvantaged participants aged 8–17 years reported that BMI between genders had significant differences after 9 years of age, and in the sit and reach test, girls performed better than boys. In Limpopo province, a study conducted by Amusa et al. (2011) with 409 rural participants aged 6–13 years revealed that boys generally performed more superiorly than girls in PF tests requiring body movement and power. On the other hand, girls were superior to boys in the tests of flexibility (Sit and reach: boys: 25.5 ± 6.70 cm; girls: 28.10 ± 6.50 cm). Armstrong, Lambert and Lambert (2011) also reported that for sit and reach, girls showed superior results for all ages compared to boys. After investigating the PF levels and weight status of Grade 1 children (*N* = 184) from two schools in Cape Town, South Africa, Van Stryp, Africa and Duncan (2022) reported that boys performed better than girls in the standing broad jump, shuttle run and throwing, whereas girls performed better in the flexibility test.

Environmental factors are also well known to influence PF (Dobosz, Mayorga-Vega & Viciana 2015; Ortega et al. 2007). Peer et al. (2013) found a significantly higher prevalence of physical inactivity among urban South African adolescents than their rural counterparts. The authors also revealed a higher prevalence of obesity among adolescents residing in urban areas. Olagbegi et al. (2022) investigated the PF profiles of rural and urban primary school children in KwaZulu-Natal, South Africa. The researcher used 520 primary school learners aged 6–13 years. The results indicated that urban learners had higher BMI than their rural counterparts. Regarding PF, rural learners had significantly higher push-ups (per minute), sit-ups (per minute), sit and reach (cm) and VO2 max, respectively. The prevalence of low PF status was significantly higher among urban learners than among their rural counterparts. However, there seem to be limited studies in South Africa profiling and comparing PF levels for urban and rural primary school children (Olagbegi et al. 2022). Therefore, this study aimed to evaluate and compare the PF levels among rural and urban primary school children in the Eastern Cape province of South Africa.

## Study design

The researcher used a cross-sectional descriptive study design for data collection in which weight, height, BMI waist circumference, push-ups (per min), sit-ups (per min), sit and reach (cm) and VO2 max were measured. The study involved a random sample of 876 learners aged 9–14. Our study posed a major potential source of bias because of the selection of participants. Thus, the sampling method of potential participants will have affected the extent of selection bias. However, as the study was population based, the resultant sample is likely to be representative of the population, which minimises selection bias. In addition, random sampling techniques were employed on the target population, and selection bias was further minimised. The sampling procedures are explained in detail in the section below.

### Sampling and sample size determination

**FIGURE 1:**
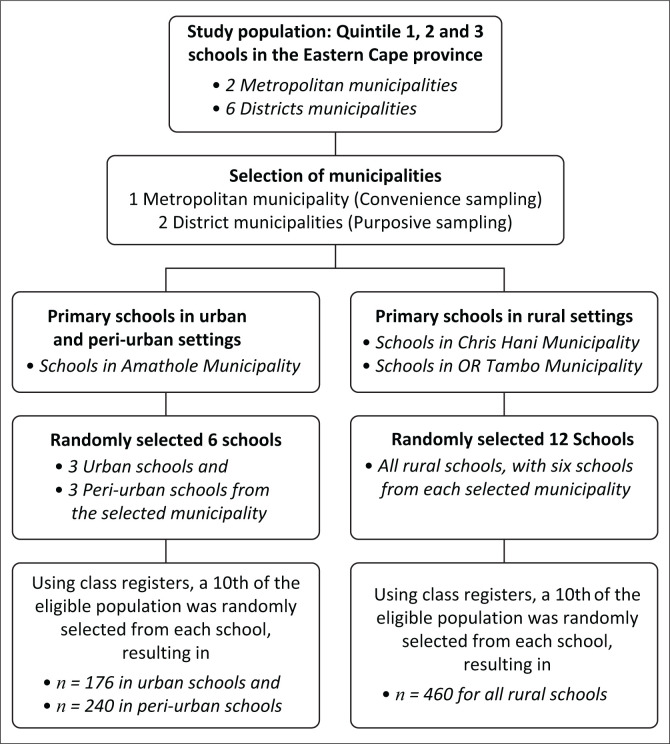
Flowchart for sampling.

Initially, out of the six provincial districts in the Eastern Cape province, one metro district municipality was conveniently sampled, and two district municipalities were purposively sampled. That is, the metro district municipality was chosen by prioritising urban settings closer to the hosting institution, while the two district municipalities were purposively chosen because of the vast number of rural primary schools within these districts. From these municipalities, a total of 18 primary schools were selected at random. Thus, schools were first arranged and numbered in alphabetical order using a database of all primary schools (obtained from the Department of Education in the Eastern Cape province) in the metro district municipality and the two district municipalities. Schools were clustered or grouped according to the respective metro and district municipalities. In each cluster, six random numbers were generated resulting in 18 primary schools being selected for the study. A 10th of the eligible population was used. Using a tenth of the eligible population as a sample size was an arbitrary choice based on the available resources, time and logistical constraints. We chose this approach with full confidence that the resultant sample would still fulfil the required sample size and provide meaningful insights while being manageable within our limitations. In each randomly selected school, grades 5 to 7 were purposively chosen. The teachers were asked to provide the class registers for each class. Learners were numbered alphabetically in their surnames (primary school registers are always numbered in this order). A pre-determined sample was established for each class, that is, 10% of the total learners. The sample was selected by choosing even numbers from the numbered class register list. Once the predetermined number was reached, the chosen learners were given a consent form to sign by a parent or guardian. This was repeated for each class (Grades 5 to 7) so that each school could have a 10th of the eligible population randomly selected from each school. Below is a flowchart summarising how sampling was conducted (see Figure 1) (Gomwe, Phiri & Marange 2023).

## Measures

### Demographic data

The participants were asked to provide their age, gender and residential setting, that is, rural, urban or peri-urban.

### Weight

A calibrated SECA scale was used to measure weight. School learners were wearing light clothing and bare-footed when climbing the scale, with their body weight equally distributed on both feet. The electronic scale was started at zero and calibrated using a known weight after every participant. All the procedures were followed to ensure the technique’s validity and reliable results.

### Height

A stadiometer was used to measure the height of school learners. Learners were asked to stand with their heels together, legs straight, arms to the side and head looking straight ahead. The learners placed their heels, buttocks, scapulae and the back of the head against the vertical board of the stadiometer. Just before the measurement was taken, the learners were asked to deeply breathe in, hold their breath and maintain (Gomwe, Seekoe, Goon, Lyoka & Marange 2019).

### Body mass index

The weight and height were calculated to determine the BMI. The standardised formula for weight and height (weight kg/height m^2^) was utilised to determine the BMI (Hammond 2000).

### Waist circumference

Waist circumference (WC) was measured according to standardised procedures described by Hammond (2000). A tape measure was used to measure the WC in centimetres. The measurements were taken at the narrowest area below the rib cage and above the umbilical cord as viewed from the front. Wrap the measuring tape around the child’s waist at this midpoint. The child should relax, exhale gently and stand still during the measurement. We ensure the tape is snug but not compressing the skin and should be parallel to the floor.

### Sit and reach

Flexibility was measured using the sit and reach test. The sit and reach test is the most common measure of flexibility, and it specifically measures the flexibility of the lumbar and hamstring muscles. This test is important as tightness in this area is implicated in lumbar lordosis, forward pelvic tilt and lumbar pain. This test was first described by Wells and Dillon (1952) and is now widely used as a reliable test of flexibility in children. The child places one hand on the other and reaches forward as far as possible, keeping their hands level. The movement should be smooth and not jerky. The child should reach forward along the measuring scale (on the sit and reach box) or towards the tape measure on the floor. The furthest point of the fingertips is recorded. The best of three attempts is taken as the final score. We used a sit-and-reach box; the score is read directly from the scale at the front edge of the box.

### Push-ups

Strength and endurance were measured using push-ups. According to the American College of Sports Medicine (ACSM), muscular endurance is the ability of body muscles to repeat muscle contractions over a long period without fatigue (ACSM 1991). It is a good predictor of muscle strength in PA. Starting position: the child places hands slightly wider than shoulder-width apart, keeps feet together or slightly apart for stability and straightens arms and legs, forming a plank position. They maintain a straight line from your head to heels, avoiding sagging hips or a raised buttock. In our study, we counted each participant’s total push-ups per minute.

### Sit-ups

Abdominal muscle endurance was measured using sit-ups. Learners were lying in a supine position on yoga gym mats. The learners were then asked to position their knees bent at 90°, both feet flat on the floor, hands behind the head and shoulder blades in contact with the floor. A research assistant helped stabilise the feet on the ground. The learners were then instructed to sit up, touch both knees with elbows and return to the starting position. The learners were to repeat this as many times as possible within 1 min. The test score was the number of sit-ups made in 1 min. Time was monitored with a chronograph digital stopwatch. We counted the number of sit-ups completed by each child within the time limit or the time taken to complete the set number of sit-ups (Gomwe et al. 2019).

### VO2 max

Cardiorespiratory fitness (CRF) was assessed by a validated maximal multistage 20 m shuttle-run test. It was tested according to the procedures described by FITNESSGRAM. The 20 m shuttle run test predicts maximal aerobic capacity. The test has 23 levels, and a level is a series of 20 m shuttle runs. Each level lasts 60 s, and the time between the recorded ‘bleeps’ decreases for each new level. The starting speed is normally 8.5 km/h and then increases by 0.5 km/h with each new level. School learners were familiarised with the procedure first. The results were entered as the number of laps per level to complete the 20 m shuttle-run test (Ramsbottom, Brewer & Williams 1988).

### Statistical analysis

Statistical Package for Social Sciences (SPSS) version 29 was used for data analysis. A descriptive approach was adopted to profile the demographic characteristics of respondents. The independent samples *t*-test and the one-way analyses of variance (ANOVA) tests were used to compare the various anthropometric measurements and PF characteristics across demographics.

### Ethical considerations

Ethical clearance to conduct this study was obtained from the University of Fort Hare, Ethical Clearance Certificate (No. LYO011SGOM01).

## Results

Out of the initial 876 respondents, 870 participants’ data were considered for further analysis. The six excluded cases were because of missing data and severe data entry and measurement errors. Table 1 shows that most participants were girls (*n* = 519; 59.7%). The average age was 11.0 years, with most participants (48.6%) aged 11–12 years. Rural-based learners comprised most participants (*n* = 459; 52.8%), followed by those from peri-urban areas (*n* = 235; 27.0%).

**TABLE 1:** Demographic characteristics of respondents (*N* = 870).

Characteristic	*n*	Valid %	Mean ± s.d.
**Gender**			
Boys	351	40.3	-
Girls	519	59.7	-
**Age (years)**			11.0 ± 1.5
**Age categories**			
9–10	222	25.5	-
11–12	423	48.6	-
13–14	225	25.9	-
**Residence**			
Urban	176	20.2	-
Peri-Urban	235	27.0	-
Rural	459	52.8	-

s.d., standard deviation.

**FIGURE 2:**
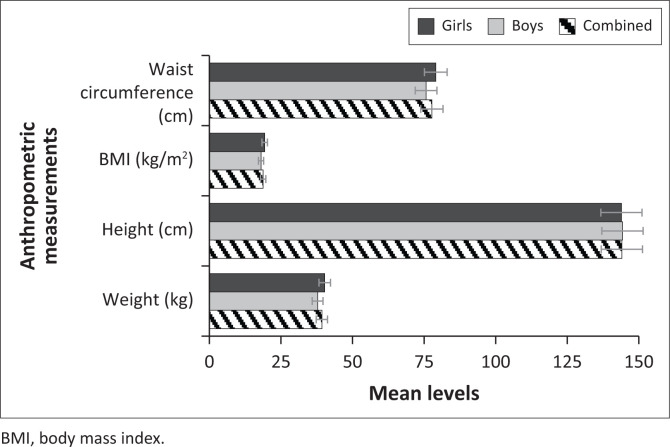
Mean levels for anthropometric measurements by gender.

**FIGURE 3:**
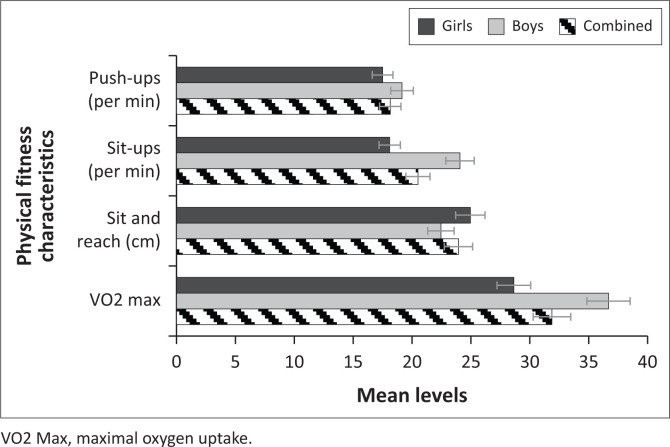
Mean levels for physical fitness characteristics by gender.

In Table 2, we have summary results for the mean levels for the anthropometric measurements and PF characteristics by gender, as well as their respective independent samples’ *t*-test *p*-values for mean comparisons. According to the findings, the average weight and height of the sample were 39.29 kg (standard deviation [s.d.] = 10.34) and 144.06 cm (s.d. = 10.81), respectively, with a mean BMI of 18.80 kg/m^2^ (s.d. = 4.11). The learners had on average, 77.72 cm (s.d. = 9.85) of waist circumference, with girls having significantly larger waist circumference (*p* ≤ 0.001). The independent samples *t*-test test also revealed that, on average, girls significantly weigh more than boys (*p* ≤ 0.001), and as a result, they had a significantly higher BMI (*p* ≤ 0.001). Regarding PF characteristics, the average push-ups and sit-ups were 18.15 per minute (s.d. = 9.41) and 20.50 per minute (s.d. = 13.23), respectively. On average, the sample had 24.06 cm (s.d. = 7.37) for the sit and reach activity, while VO2 max had a mean level of 31.88 (s.d. = 10.23). The independent samples *t*-test test shows that the mean levels for push-ups, sit-ups and VO2 max were higher in boys than girls. On the contrary, the independent samples *t*-test revealed that the mean levels for sit and reach were significantly higher (*p* ≤ 0.001) in girls (24.95 ± 7.49) as compared to that of boys (22.74 ± 7.86). Figure 2 and Figure 3 show the graphical comparisons for the anthropometric measurements and PF characteristics by gender.

**TABLE 2:** Summary mean levels with respective independent samples *t*-test mean comparisons for anthropometric measurements and physical fitness characteristics by gender.

By gender	Combined (*N* = 870) Mean ± s.d.	Boys (*N* = 351) Mean ± s.d.	Girls (*N* = 519) Mean ± s.d.	*p*
**Anthropometric measurements**				
Weight (kg)	39.29 ± 10.34	37.81 ± 8.53	40.29 ± 11.30	< 0.001*
Height (cm)	144.06 ± 10.81	144.29 ± 10.40	143.91 ± 11.08	0.625
BMI (kg/m^2^)	18.80 ± 4.11	18.04 ± 2.98	19.32 ± 4.65	< 0.001*
Waist circumference (cm)	77.72 ± 9.85	75.69 ± 8.41	79.08 ± 10.50	< 0.001*
**Physical fitness characteristics**				
Push-ups (per min)	18.15 ± 9.41	19.15 ± 10.29	17.50 ± 8.71	0.013*
Sit-ups (per min)	20.50 ± 13.23	24.07 ± 13.50	18.10 ± 12.49	< 0.001*
Sit & reach (cm)	24.06 ± 7.37	22.74 ± 7.86	24.95 ± 7.49	< 0.001*
VO2 max	31.88 ± 10.23	36.68 ± 11.20	28.64 ± 8.05	< 0.001*

BMI, body mass index; s.d., standard deviation; VO2 Max, maximal oxygen uptake.

* , *p*-value < 0.05 - Statistically significant differences.

**FIGURE 4:**
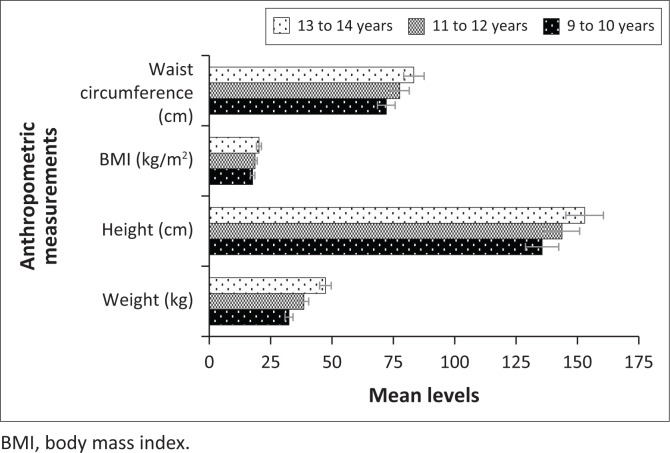
Mean levels for anthropometric measurements by age.

**FIGURE 5:**
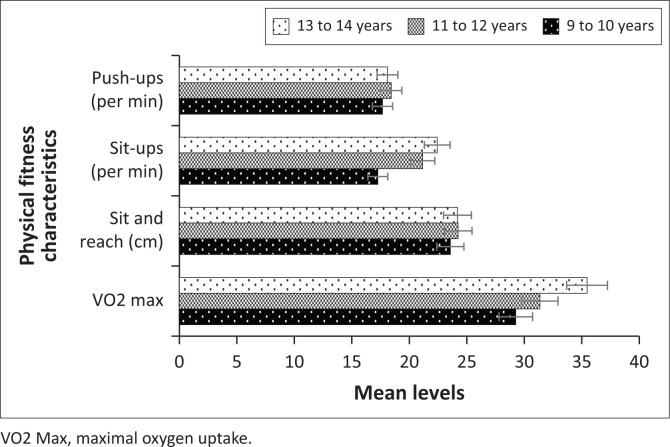
Mean levels for physical fitness characteristics by age.

The summary of the mean levels for the anthropometric measurements and PF characteristics by age is presented in Table 3. Assessing the anthropometric measurements, the one-way ANOVA test and respective post-hoc pairwise comparisons showed that weight, height, BMI and waist circumference significantly increase with age (all *p* ≤ 0.001). On the other hand, there are no statistically significant differences among the various age groups regarding the mean levels of push-ups (*p* = 0.621) and sit and reach (*p* = 0.527). On the contrary, the mean sit-up levels were significantly higher in older children than the younger children (*p* ≤ 0.001). Similarly, the results revealed that the mean levels for VO2 max significantly increased with age (*p* ≤ 0.001). Figure 4 and Figure 5 show the graphical comparisons of the anthropometric measurements and PF characteristics by age.

**TABLE 3:** Summary mean level comparisons for anthropometric measurements and physical fitness characteristics by age.

Variable	9 to 10 years (*n* = 222) Mean ± s.d.	11 to 12 years (*n* = 423) Mean ± s.d.	13 to 14 years (*n* = 225) Mean ± s.d.	*p*
**Anthropometric measurements**				
Weight (kg)	32.50 ± 7.18^c^	38.58 ± 8.58^b^	47.32 ± 10.66^a^	< 0.001*
Height (cm)	135.64 ± 8.60^c^	143.74 ± 8.61^b^	152.97 ± 9.50^a^	< 0.001*
BMI (kg/m^2^)	17.67 ± 3.74^c^	18.63 ± 3.78^b^	20.25 ± 4.61^a^	< 0.001*
Waist circumference (cm)	72.15 ± 8.94^c^	77.62 ± 8.29^b^	83.39 ± 10.26^a^	< 0.001*
**Physical fitness characteristics**				
Push-ups (per min)	17.67 ± 9.04	18.43 ± 9.29	18.10 ± 9.41	0.621
Sit-ups (per min)	17.26 ± 11.30^b^	21.16 ± 13.47^a^	22.43 ± 13.98^a^	< 0.001*
Sit & reach (cm)	23.57 ± 6.71	24.24 ± 7.49	24.19 ± 7.76	0.527
VO2 max	29.26 ± 8.73^c^	31.36 ± 9.90^b^	35.46 ± 11.23^a^	< 0.001*

BMI, body mass index; s.d., standard deviation; VO2 Max, maximal oxygen uptake.

* , *p*-value < 0.05 - Statistically significant differences.

a,b,c , Grouping for post-hoc pairwise comparisons, where (a,b,c) represents statistically significant different mean levels.

**FIGURE 6:**
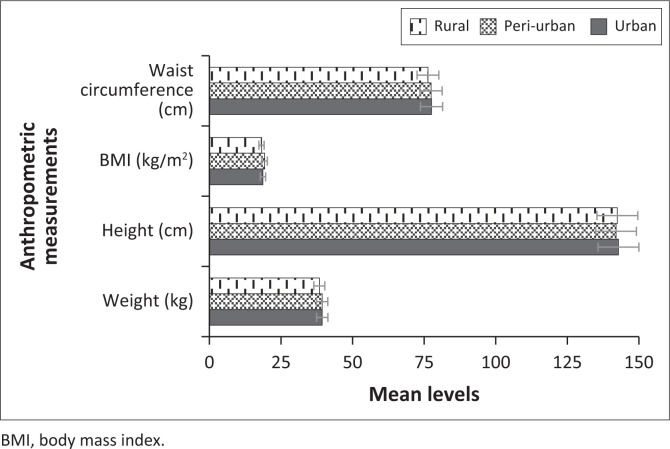
Mean levels for anthropometric measurements by residence.

**FIGURE 7:**
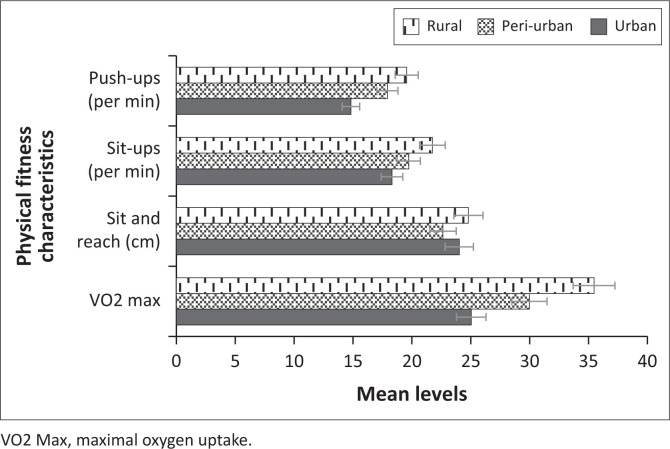
Mean levels for physical fitness characteristics by residence.

Lastly, assessing the mean levels for the anthropometric measurements and PF characteristics by place of residence (see Table 4), the one-way ANOVA test showed that weight, height and waist circumference were not significantly different by place of residence (all *p* > 0.05). Only BMI showed significant differences by residence (*p* = 0.004), whereas learners from peri-urban and urban areas had higher levels of BMI. On the other hand, the results showed statistically significant differences in place of residence concerning the mean levels of push-ups (*p* ≤ 0.001). The grouping for the Waller–Duncan post-hoc pairwise comparisons reveals that those from rural areas (19.56 ± 8.76) had significantly higher mean levels for push-ups followed by those from peri-urban areas (17.92 ± 10.35) with those from urban areas having the least mean levels of push-ups. The mean levels for sit-ups were significantly higher for children from rural areas (21.74 ± 13.76) as compared to those from peri-urban areas (19.73 ± 12.41) and urban areas (18.31 ± 12.57). The results also revealed that the mean levels for VO2 max were significantly higher in children from rural areas (35.47 ± 11.10), while those from urban areas (25.04 ± 6.45) had the lowest mean levels of VO2 max. Regarding sit and reach, children from urban and rural areas had significantly higher mean levels than those from peri-urban areas (*p* = 0.001). Figure 6 and Figure 7 show the graphical comparisons of the anthropometric measurements and PF characteristics by place of residence.

**TABLE 4:** Summary mean level comparisons for anthropometric measurements and physical fitness characteristics by residence.

By residence	Urban (*n* = 176) Mean ± s.d.	Peri-Urban (*n* = 235) Mean ± s.d.	Rural (*n* = 459) Mean ± s.d.	*p*
**Anthropometric measurements**				
Weight (kg)	39.84 ± 10.18	39.80 ± 10.15	38.81 ± 10.49	0.361
Height (cm)	144.44 ± 9.19	143.59 ± 12.65	144.06 ± 10.81	0.702
BMI (kg/m^2^)	18.89 ± 3.47^a,b^	19.50 ± 5.73^a^	18.41 ± 3.19^b,c^	0.004*
Waist circumference (cm)	78.43 ± 9.49	78.25 ± 9.35	77.17 ± 10.21	0.217
**Physical fitness characteristics**				
Push-ups (per min)	14.82 ± 8.87^c^	17.92 ± 10.35^b^	19.56 ± 8.76^a^	< 0.001*
Sit-ups (per min)	18.31 ± 12.57^b^	19.73 ± 12.41^b^	21.74 ± 13.76^a^	0.008*
Sit and reach (cm)	24.02 ± 5.83^a^	22.63 ± 6.79^b^	24.80 ± 8.05^a^	0.001*
VO2 max	25.04 ± 6.45^c^	29.98 ± 7.24^b^	35.47 ± 11.10^a^	< 0.001*

BMI, body mass index; s.d., standard deviation; VO2 Max, maximal oxygen uptake.

* , *p*-value < 0.05 - Statistically significant differences.

a,b,c , Grouping post-hoc pairwise comparisons, where (a,b,c) represents statistically significant different mean levels.

## Discussion

This research study looked at the effects of the place of residence on PF levels and body composition of school learners. The results indicated that school learners from different residences differ significantly in PF and body composition. On average, girls weigh significantly more than boys (*p* = 0.006), and as a result, they had a significantly higher BMI (*p* ≤ 0.001). These established gender differences are supported by Travill (2007), who reported that the BMI of boys and girls had significant differences. The reason could be that girls are more protected (i.e., more restricted in movement, e.g., visiting friends, going to social gatherings, etc.) than boys in both urban and rural residences, so their physical activities to lose weight are restricted (Sylejmani et al. 2019). Further, because of historical gender roles, girls are more likely to have chores and responsibilities in the home as compared to boys. On the other hand, our study also revealed that BMI significantly increases with age, and these findings are similar to most of the research studies reported in the literature (Gouveia et al. 2021; Torres-Luque et al. 2018).

The mean levels of boys in push-ups, sit-ups and VO2 max were higher than in girls. In support, in a study by Amusa et al. (2011), the authors revealed that boys performed better than girls in PF tests requiring body movement and power. The assumption is that boys have more muscles anatomically as compared to girls (Torres-Luque et al. 2018). On the contrary, girls have higher mean levels for sit and reach than boys. This is well supported in literature where several South African-based studies reported that girls are superior to boys in the tests of flexibility (Amusa et al. 2011; Gouveia et al. 2021; Travill 2007; Van Stryp et al. 2022). The reason why girls perform better for sit and reach (flexibility) is that girls are more flexible than boys (Gomwe et al. 2022). Our results also suggest that sit-up levels were significantly higher in older than younger children. In addition, the mean levels for VO2 max significantly increased with age. Thus, during adolescence, muscles increase with age. Therefore, older learners can perform more than young learners. This supports a study by Torres-Luque et al. (2018), which demonstrated that age influences fitness measurements.

Learners from rural areas had significantly higher mean levels for push-ups, sit-ups and VO2 max than those from urban and peri-urban areas. Similar tendencies have been determined for fitness and place of residence, where rural learners have reported significantly higher push-ups (per minute), sit-ups (per minute) and VO2 max (Olagbegi et al. 2022; Torres-Luque et al. 2018). A probable explanation for rural school learners’ better PA performance could be that school learners living in rural areas may have more active opportunities than urban peers because rural learners walk a long distance to and from school (Craig et al. 2013). They have less access to social media and other modern technologies. These factors could have enhanced rural school learners’ potential for increased PA performance and helped reduce their sedentary behaviour (Craig et al. 2013). Although there are more free public PA facilities in urban areas than rural areas, the lack of safety and high crime rates make it difficult for urban school learners to participate in PA (Gomwe et al. 2022). Our results also showed that in terms of sit and reach, children from rural areas had higher mean levels but were not significantly different from those from urban areas (*p* = 0.140). The reason could be that flexibility is more dependent on gender than environmental factors such as place of residence.

In general, the findings from sub-Saharan Africa contradict the results from the developed countries (Hian et al. 2013). The probable reason could be the environmental factors. Most developed countries are urbanised as compared to developing countries. South Africa is still moving towards urbanisation. These findings suggest that with further urbanisation, the prevalence of non-communicable diseases may increase if appropriate intervention programmes are not implemented.

### Limitations of the study

The major limitation of this study might be human measurement error, even though the research assistants had extensive prior training. In addition, the study was not conducted in a controlled environment such that food consumed by participants before the fitness exercises might have disadvantaged or advantaged some participants over others.

## Conclusion

Based on the findings obtained, the present study concluded that rural school learners had better performance in push-ups (per min), sit-ups (per min), sit and reach (cm) and VO2 max as compared to their urban counterparts. The effect of place of residence on PF of rural and urban primary school learners should be considered when designing future intervention programmes. There is also a need to encourage PF in children and reintroduce formal physical education into the South African school curriculum. Further, there is a need for theory-based interventions (Hereen et al. 2017) that are meant to effectively increase self-reported adherence to PA guidelines among primary school learners in South Africa.
